# 

**Published:** 1909-04

**Authors:** 


### BOOKS RECEIVED.

Primary Studies for Nurses: A Textbook for First Year Pupil Nurses. By Charlotte A. Aikens, formerly Superintendent of Columbia Hospital, Pittsburg, and of the Iowa Methodist Hospital, Des Moines. 12 mo. of 435 pages, illustrated. Philadelphia and London: W. B. Saunders Company, 1909. Cloth, $1.75 net.

Practical Dietetics with reference to Diet in Disease, by Alida Frances Pattee, graduate, Boston Normal School of Household Arts. Late Instructor in Dietetics, Bellevue Training School for Nurses, Bellevue Hospital, New York City. Fifth edition. 12 mo, cloth. 300 pages. A. F. Pattee, Publisher, Mt. Vernon, N. Y. New York Office, 52 West 39th St., N. Y. (Price, $1.00 net. By mail, $1.10. C. O. D. $1.25.)

Refraction and How to Refract, including sections on Optics, Retinoscopy, fitting of Spectacles and Eye Glasses. By James Thorington, A.M., M.D., professor of diseases of the eye in the Philadelphia Polyclinic and College for graduates in medicine, etc. Fourth edition. Two hundred and twenty illustrations, thirteen being in colors. Philadelphia: P. Blakiston's Son & Company. 1909. (Price, cloth $1.50.)

Parcimony in Nutrition, by Sir James Crichton-Browne, M.D., LL.D., F.R.S. 12 mo. Funk & Wagnalls Company, New York. (Cloth, 75 cents, net.)

Report of the United States Commissioner of Education, for the year ended June 30, 1908. Volume 1. Washington Government Printing Office. 1908.

Theory and Practice of Infant Feeding with notes on development, by Henry Dwight Chapin, A.M., M.D. Professor of Diseases of Children. New York Post-Graduate Medical School and Hospital. Third edition, revised, with illustrations. New York: William Wood & Co. 1909.

New and Nonofficial Remedies. Articles which have been accepted by the Council on Pharmacy and Chemistry of the American Medical Association, prior to January, 1909. Chicago: Press of the American Medical Association, 103 Dearborn Avenue, (Paper, 25c; cloth, 50c.)

Epoch-Making Contributions to Medicine, Surgery, and the Allied Sciences; being reprints of those communications which first conveyed Epoch-Making observations to the scientific world, together with biographical sketches of the observers. Collected by C. M. B. Camac, M.D., of New York City. Octavo of 435 pages, with portraits. Philadelphia, London. W. B. Saunders Company, 1909. Artistically bound, $4.00 net.

A Textbook of Medical Chemistry and Toxicology. By James W. Holland, M.D., Professor of Medical Chemistry and Toxicology, Jefferson Medical College, Philadelphia. Second revised edition, octavo of 655 pages, fully illustrated. Philadelphia and London: W. B. Saunders Company, 1908. (Cloth, $3.00 net.)

A Textbook of Materia Medica, Pharmacology and Therapeutics. By George F. Butler, M.D., Professor and Head of the Department of Therapeutics and Professor of Preventive and Clinical Medicine, Chicago College of Medicine and Surgery, Medical Department Valpariso University. Sixth edition, revised and enlarged. Octavo of 708 pages. Philadelphia and London: W. B. Saunders Company, 1908. (Cloth, $4.00 net, half-morocco, $5.50 net.)

International Clinics. A Quarterly of Illustrated Clinical Lectures and especially prepared articles on Treatment, Medicine, Surgery, Neurology, Pediatrics, Obstetrics, Gynecology, Orthopedics, Pathology, Dermatology, Ophthalmology, Otology, Rhinology, Laryngology, Hygiene and other topics of interest to students and practitioners. Edited by W. T. Longcope, M.D., Volume I. Nineteenth series. Philadelphia and London: J. B. Lippincott Co. 1908. (Cloth, $2.00.)

Progressive Medicine. A Quarterly Digest of Advances, Discoveries and Improvements in the Medical and Surgical Sciences. Edited by Hobart Amory Hare, M.D., Professor of Therapeutics and Materia Medica in the Jefferson Medical College of Philadelphia, Vol. XI, No. 1, March, 1909, Octavo, 277 pages. Per annum, in four paper-bound volumes, containing 1,200 pages. Lea & Febiger, Publishers, Philadelphia and New York: ($6.00, net; in cloth, $9.00, net.)

